# Improving draft assemblies by iterative mapping and assembly of short reads to eliminate gaps

**DOI:** 10.1186/gb-2010-11-4-r41

**Published:** 2010-04-13

**Authors:** Isheng J Tsai, Thomas D Otto, Matthew Berriman

**Affiliations:** 1 Parasite Genomics, Wellcome Trust Sanger Institute, Wellcome Trust Genome Campus, Hinxton, Cambridge, CB10 1SA, UK

## Abstract

Advances in sequencing technology allow genomes to be sequenced at vastly decreased costs. However, the assembled data frequently are highly fragmented with many gaps. We present a practical approach that uses Illumina sequences to improve draft genome assemblies by aligning sequences against contig ends and performing local assemblies to produce gap-spanning contigs. The continuity of a draft genome can thus be substantially improved, often without the need to generate new data.

## Abstract 2

IMAGE generates local assemblies, closing gaps in genomes assembled from paired-end next generation sequencing data, often without the need for new data

## Background

The complete genome sequence of an organism provides an invaluable resource to the wider research community and is the foundation for comparative and evolutionary genomics studies. With the recent advances in second-generation sequencing technologies (454 pyrosequencing, Illumina, SOLiD, and Helicos), genome projects have seen an explosion of sequence data production at a fraction of the per-base cost. However, this cost reduction is compromised by typically shorter sequence lengths, and unique profiles of sequencing errors compared with conventional capillary reads [1]. This leads to new computational challenges in assembly to address each of these differences as well as subsequent downstream analyses.

The performance of *de novo *assembly software depends heavily on the sequence length, depth of sequence coverage (genome equivalents, or fold coverage), fragment size of the templates that are sequenced and the types of sequence errors specific to each technology. The situation is complicated by the range of assembly software that exists for use with second-generation technologies. For example, Newbler, produced by Roche, specifically addresses 454 read-specific error profiles. A range of assemblers are available for *de novo *assembly of Illumina reads, including Velvet [2], Abyss [3], SOAPdenovo [4] and ALLPATHS2 [5], each of which is designed with a different aim and functionality. As second-generation sequencing technologies are improving at different paces, both in error rate and sequence length, assembling a mixture of sequences from different technologies remains a viable strategy for sequencing genomes *de novo*.

Currently, few assemblers (for example, Newbler and Velvet) are able to incorporate mixtures of read types, and their accuracy remains to be assessed. An alternative approach is to combine sequence information from different technologies by using bioinformatics pipelines to assemble contigs from each sequencing technology separately, before treating them as *faux *reads in a combined assembly to further scaffold and close gaps [6,7]. The final consensus sequences created in this way are mosaics of the contig sequences generated from each of the component sequencing technologies. This makes it difficult to assess accuracy as relationships between reads and contigs are typically lost during intermediate stages of these pipelines.

Draft genome assemblies vary in their quality [8]. A highly accurate genome sequence reduces the time needed to distinguish results of real biological interest from artifacts due to misassemblies. For the human genome [9], the draft assembly was followed by a labor-intensive finishing phase where the assembled sequences were improved using targeted sequencing to resolve misassembled regions, close sequence gaps, and improve coverage and accuracy in sparsely covered regions of the genome. Misassemblies and gaps usually result from repeats, as well as secondary structures, underrepresented GC-rich regions or regions simply not sequenced due to a low depth sequence coverage [10].

The standard strategy to close gaps usually involves the design of specific oligonucleotide primers to undergo semi-automated targeted sequencing at contig ends [11,12]. Reads are extended and manually aligned to close gaps and resolve questionable regions. Although contiguation is improved in this way, the process is labor intensive and time consuming and, as a result, expensive. The massive increases in data volumes and the small contig sizes associated with second generation sequencing data further increase the time and costs needed to advance a genome from a draft assembly to an improved or finished state [8].

In this study we have developed an approach - called Iterative Mapping and Assembly for Gap Elimination (IMAGE) - to raise the quality of draft assemblies towards finished, but without manual intervention, using local assemblies of reads from gap regions. The approach utilises the large number of sequences that an Illumina Genome Analyzer produces. Reads that correspond to gaps or questionable regions are identified and reassembled locally before being incorporated back into the final assembly. An advantage of a local assembly as opposed to a *de novo *one is that the number of reads used is only a fraction of total available reads. This reduces the complexity of regions to be assembled as well as the time and computing memory required. We demonstrate each stage of our approach and show the reassembled region can reach up to 10 kb in a simulated dataset. We demonstrate the improvement of this approach in assemblies of any read types in several ongoing genome projects up to 350 Mb.

## Results

**Figure 1:**
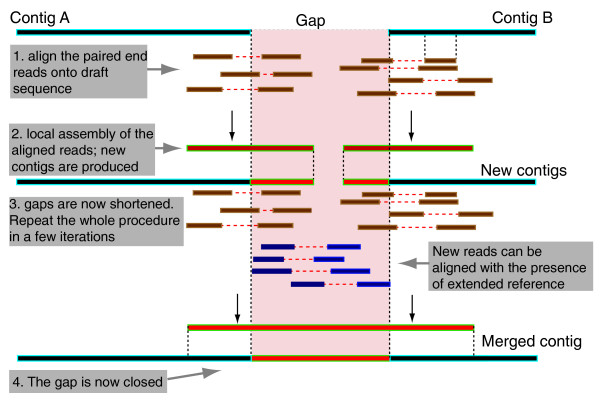
**Overview of the IMAGE process**. Step one, Illumina reads are aligned against the initial assembly. Step two, Illumina reads that align to contig ends, along with their non-aligning mate adjacent to gaps, are assembled into new contigs, which are subsequently mapped back to the initial assembly. Step three, Illumina reads are aligned against the updated assembly and the whole process is repeated iteratively until the gap is closed.

We used IMAGE to improve genome assemblies by targeted re-assembly of Illumina reads to span gaps between adjacent contigs within scaffolds. As illustrated in Figure 1, the approach aligns and gathers Illumina reads at the ends of contigs and performs a local assembly of these reads to produce new contigs. The newly assembled Illumina contigs are used to extend or merge existing contigs within a reference, before iterating the whole process to perform further walks into gaps.

### Assessment of local assemblies in a simulated dataset

To evaluate different stages of the pipeline, we applied IMAGE to three simulated assemblies that we produced from the previously finished 4.6 Mb genome sequence of the enterobacteria *Salmonella enterica *serovar Paratyphi. Each assembly contained contigs of 30 kb in length, which were separated by gaps of fixed length (1, 2 or 10 kb). Simulated Illumina reads of 76 bp from either end of 300 bp fragments and with a depth of coverage of approximately 25-fold were also generated from the reference sequence. The contigs and reads were exactly the same as the original genome sequence, and the positions of the contigs and Illumina reads relative to the reference sequence were recorded so that the performance could be assessed in various ways.

We applied our algorithm to the simulated datasets and found that, on average, 94.3% (379 out of 402) of gaps could be closed correctly irrespective of gap size, with 100% identity (Table 1). This implies that the pipeline can achieve high accuracy if the quality of the Illumina sequences is high, in this case containing no errors. The complexity of the genome in question is also a contributing factor and, in this case, bacterial genomes have very few repeats. Only 4 out of 383 closed gaps were misassembled, most of them being the extremely long 10 kb gaps.

**Table 1:** Assessment of closed gaps in the simulated assembly of *Salmonella paratyphi*

Introduced gap length (kb)	Fate of gaps	
	
	Fraction closed with 100% identity	False rate^a^
1	0.99 (145/147)	0 (0/145)
2	0.96 (136/142)	0.007 (1/137)
10	0.90 (102/113)	0.029(3/105)

^a^The false rate was determined by finding the number of misassemblies over number of closed gaps.

There were two main causes for misassemblies in the new inserted contigs or unclosable gaps. Either, the sequences that fell into the gaps were repetitive or the sequences flanking the gap were repetitive. In the first case, assembly of repetitive regions using short reads is challenging [2] because any parameters of the assembly algorithm that have been optimised for a whole genome assembly may not necessarily perform well for a subset of reads. Although all of the reads necessary to assemble these gaps were successfully pooled, Velvet was unable to reassemble the region correctly despite using a variety of parameters. The second situation occurred with both of the unclosed 1 kb gaps in the simulated dataset (Table 1). The sequences at these contig ends were present at least six times in the genome; thus, no reads were available at the assembly stage to close them.

### Assembly improvement in seven pathogen genomes

We attempted to improve draft assemblies of seven ongoing genome sequencing projects in the Pathogen Genomics group of Wellcome Trust Sanger Institute, which consisted of capillary, 454 and Illumina paired end (PE) reads at various levels of coverage. We then applied IMAGE to these assemblies with Illumina PE reads with successive iterations until no more gaps closed. The summary of the improved assemblies is shown in Table 2. In each case, approximately 50% of gaps that occurred within scaffolds were closed and contig lengths were increased as a result of original contigs being merged by Illumina contigs. The general performance of IMAGE varied with different lengths of fragment size and the lengths of Illumina reads themselves rather than the coverage of the sequenced reads. For example, 55% of *Schistosoma mansoni *gaps were closed with only approximately 30-fold coverage of Illumina PE reads available.

**Table 2:** Description of assemblies, Illumina reads and performance of IMAGE

	Statistics of draft assembly			Illumina reads used			IMAGE performance	
	
Organisms	Read type	Size (Mb)	N50^a^(kb)	Read length	Coverage^b^	Insert size^c^	Total gaps^d^	Gaps closed
*Salmonella enterica *1	454	4	117	54	295	225	73	49 (68%)
*Salmonella enterica *2	Illumina	4	87	54	274	220	233	194 (83%)
*Clostridium difficile*	454	4	76.5	54	331	249	118	55 (47%)
*Bordetella bronchiseptica*	454	5	5	54	420	321	918	397 (43%)
*Plasmodium berghei*^e^	Cap+454+Illumina	18.5	189	76	140	130	156	71 (46%)
*Leishmania donovani*	454	30	10	76	83	176	3,826	1,768 (46%)
*Echinococcus multilocularis*	Cap+454	107	108	75	126	310	1,676	895 (53%)
*Schistosoma mansoni*	Cap	307	24	108	30	219	25,214	13,771 (55%)

^a^The minimum contig length cutoff to include contigs in a given assembly to have 50% of total assembled sequence. ^b^Estimated coverage is calculated as Length of Illumina read × Number of reads/Assembled genome size. ^c^Insert size is defined as the averaged physical distance between two sequenced fragments that were unambiguously aligned against the contig sequence of the initial assembly. ^d^Gap is defined as the region between contigs within a scaffold that has no sequence information. ^e^The core set of *Plasmodium berghei *contigs that were aligned to the closely related reference sequence of *Plasmodium chabaudi*. Cap, capillary reads.

Next, we identified three use cases to assess the practicability and performance of IMAGE in improving a *de novo *assembly of mixed 454 and capillary sequencing data, a guided assembly (that is, of scaffolds from a comparative genomics study) or a *de novo *assembly from Illumina sequencing reads only.

### Improving *de novo *assembly from capillary/454 reads

In the first case study, the original draft assembly of the tapeworm *Echinococcus multilocularis *determined by whole-genome shotgun capillary and 454 sequencing was improved using Illumina reads with a depth of coverage of approximately 120-fold. Illumina reads were firstly aligned to the draft assembly; 81.4% of the read-pairs mapped with at least one mate unambiguously aligned. About 5% of the total reads were found less than 600 bp away from contig ends. These reads were gathered along with their mate reads and partitioned into sets according to the contig ends to which they were mapped. Scaffolding information was used to assemble Illumina reads that span the same gap. Most sets of reads were assembled into single contigs of various lengths and could be aligned back to the original contigs. Where they contained lower quality sequence, insertions or deletions, the contig ends from the original assembly could be corrected by the new contigs, thus enabling more Illumina reads to be aligned in subsequent iterations.

**Figure 2:**
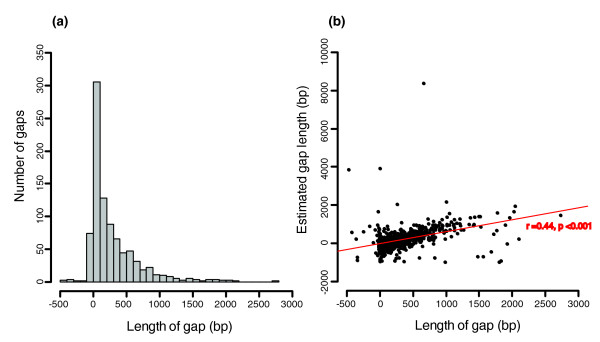
**Statistics of sequences at closed gaps in the *Echinococcus multilocularis *assembly**. **(a) **The frequency of length of newly inserted sequences at gaps. **(b) **The closed gap length is positively correlated with estimated gap length from the Arachne assembler (Pearson's r = 0.44, *P *< 0.001).

In total, 895 out of 1,676 sequence gaps were closed by IMAGE, which was run for 9 iterations - until no more gaps could be closed - though 662 gaps closed in the first iteration. In contrast, a *de novo *assembly of the entire set of Illumina reads results in far fewer gaps being closed (116 gaps; Additional file 1). The new sequences were approximately 372 kb in total and were inserted into the initial assembly. The number of contigs in the improved assembly was reduced to just 1,414, with the largest contig 1.6 Mb in length. Examination of the distribution of closed gap lengths showed that they were typically approximately 50 bp (Figure 2a), and were significantly correlated to the estimated gap size produced by Arachne [13] using the read insert length (Pearson's r = 0.44, *P *< 0.001; Figure 2b). The largest closed gap was 2,733 bp and contained a 90 bp stretch of the *raf *gene encoding the *E. multilocularis raf *serine kinase. There were 78 closed gaps with negative lengths, indicating that the spanning gap was artificial and that the contig pairs bridged by these gaps could be joined without additional sequencing.

To investigate the relative quality of contig ends and closed gaps in *E. multilocularis*, we focused on the 818 true gaps, that is, not those with negative lengths. Of these, 524 had Illumina contigs that mapped exactly to the initial assembly, indicating that these gaps could be closed with high confidence. We manually inspected some of the gaps where the newly inserted sequences had discrepancies with the initial assembly, usually identified as significant mismatches by SSAHA2. We defined these discrepancies as 'overhangs' in the initial assembly, usually due to low quality bases at the ends of capillary sequences that form part of a consensus sequence. The overhangs result in some unclosable gaps, which IMAGE addresses by identifying them and replacing them with Illumina contigs. As a result, more Illumina reads can be aligned against the contig ends in subsequent iterations.

We designed oligonucleotides to confirm, by PCR, that 100 randomly chosen gaps had been correctly closed by IMAGE. PCR products were obtained from 97 reactions and were sequenced. After quality screening the sequences for 71 gaps were obtained. In all but one case the sequenced PCR product matched the in-filled gap sequence obtained by IMAGE. The cause of the only discrepancy was 30 copies of a GAA repeat (90 bp). This repetitive region was longer than the 76 bp length of an Illumina read and therefore difficult for Velvet to assemble, resulting in a contig that only spanned 24 bp of GAA.

### Closing gaps in a guided assembly

**Figure 3:**
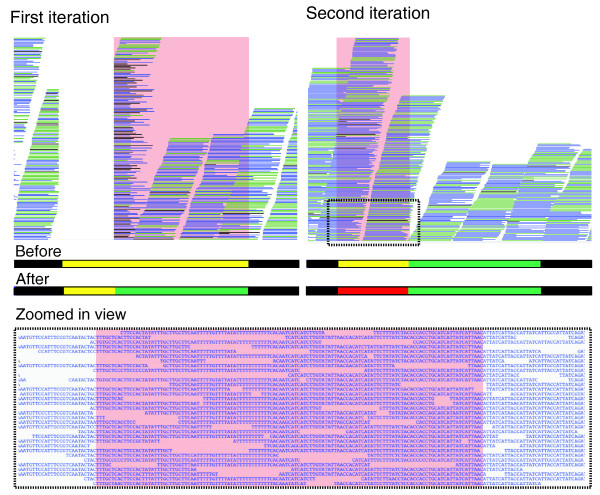
**An example of a gap closed with two iterations of IMAGE in *Plasmodium berghei***. In the first iteration, IMAGE extended the contig consensus sequence from the right side of the gap, indicated by the green bar. In the second iteration, reads were aligned to the updated contig end. Local assembly of these reads along with their unaligned mates resulted in a new contig to completely close the gap, indicated by the red bar. The horizontal lines above the bars denote the Illumina reads realigned to the updated consensus sequence after each iteration. Below, a zoomed in plot shows the Illumina reads realigned against the closed gap.

In our next case study, we used Illumina reads to improve the assembly of the genome of the malaria parasite *Plasmodium berghei*, which had been determined by aligning and orientating contig sequences from various technologies (capillary, 454 and Illumina) against its close relative *Plasmodium chabaudi *(see Materials and methods). IMAGE was run until no further gaps could be closed (3 iterations), closing 71 out of 156 gaps. Curiously, these gaps were closed with the same reads that were used to generate the Illumina contigs of the initial assembly. It remains to be determined why the assembly algorithms used to generate the initial assembly (Phusion [14], Velvet) failed to close these regions. As shown in Figure 3, closer inspection of the gap reveals slightly higher than average coverage of Illumina reads aligned against the new sequence at gaps compared to the rest of the reference.

### Improving an assembly comprising only Illumina reads

Based on the results seen with the *P. berghei *assembly, we sought to examine whether the same sets of Illumina reads could be used to produce a *de novo *assembly and subsequently improve it using IMAGE. As IMAGE gathers uniquely mapped reads at contig ends, their unmapped mates, which velvet could not utilize in a *de novo *setting, can be reused to close gaps using a range of different parameters. A draft genome assembly was produced by Velvet from Salmonella enterica using solely Illumina 54 bp reads. The assembly contained 233 gaps and 12 iterations of IMAGE were run with a range of k-mer settings (see Materials and methods). As a result, a total of 194 (83%) gaps were closed from local assemblies of reads aligning to the gap regions, despite those reads having been present in the original dataset. Five of the remaining unclosable gaps contain multiple copies of ribosomal RNA genes and will remain difficult to assemble with any short read assemblers.

**Figure 4:**
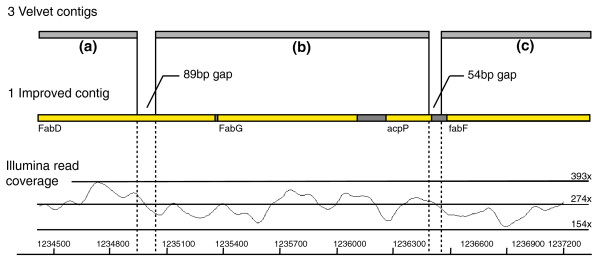
**Closing gaps in *de novo *assembly comprising only Illumina reads**. Schematic diagram showing the comparison of the original velvet assembly (3 contigs a, b and c) and the improved assembly in *Salmonella enterica*. The improved assembly was aligned to the reference sequence with 99.8% identity. The two closed gaps shown were 100% identical to the reference sequence. Contigs are indicated by grey bars; gene annotations are indicated by yellow boxes. Vertical lines highlight the gaps that are filled by IMAGE in the improved contigs. Below, a coverage plot showing the relatively even depth of coverage of realigned Illumina reads at the improved assembly, indicating no signature of misassembly.

To evaluate the accuracy of the closed gaps, we aligned the original and improved assembly against the finished sequence of *S. enterica*. As illustrated by Figure 4, the improved assembly and the reference sequence are in good agreement without obvious signatures of misassembly. Next, we realigned Illumina reads against the updated assembly using SSAHA2 and assessed the coverage depth at closed gaps. The coverage plot depicted in Figure 4b shows no obvious discrepancies of coverage at closed gaps compared to the rest of the sequence.

## Discussion

Assemblies produced from any sequencing technology produce gaps irrespective of the assembler used, mainly due to sampling biases in the library preparation or repetitive regions. There is, therefore, an urgent need for tools to efficiently improve the draft assemblies in an automated fashion so that draft genomes are more accurate and contiguous without the additional cost of manual intervention. IMAGE simplifies the assembly problem by targeting specific regions (gaps), which reduces both the time and computational resources needed. IMAGE performed well across a range of real genome assemblies; we were able to close around half of targeted gaps. All of the draft assemblies used in this study were improved from a modest coverage of Illumina sequences, as low as 30× in the case of *Schistosoma mansoni*. Few gaps were misassembled, but in most cases they were large and could be readily detected as misassemblies by assessing their depth of coverage with realigned Illumina reads. For example, a misassembled (collapsed) repeat region will have an unusually high depth of coverage.

With the increasing availability of next-generation sequencing technologies, one of the main motivations behind IMAGE was to improve existing assemblies using additional data from Illumina sequencing. In the first case study, more than half of the gaps in a draft *Echinococcus *genome could be closed using IMAGE without the need for replacing the original assembly with a new one assembled *de novo*. In fact, we also showed that a *de novo *assembly of the Illumina data provides less information compared with incorporating the data using IMAGE and is far more computationally expensive.

Using local assemblies to resolve problematic regions is not a new idea; it is commonplace during manual finishing but is laborious and slow. Local assemblies have also recently been implemented in SOAPdenovo to resolve repeats in a *de novo *assembly [4]. In the latter case, Illumina reads are assembled *de novo *but repetitive regions are masked during the scaffolding process and these regions (subsequently referred to as 'gaps') are then resolved by reassembly of appropriate existing PE reads. IMAGE differs from this approach in two ways: it establishes new linkage information between the newly sequenced reads and any existing assembly by mapping, and then improves the assembly by localised assembly of reads at real gaps and regions that were previously unresolved in the assembly; and it uses iterative rounds of gathering reads and reassembles them to span gaps to close large intra-scaffold gaps that are longer than the fragment size of the sequencing library.

We also demonstrated that IMAGE can close gaps between contigs from the same set of Illumina reads that were used to generate the original *de novo *assembly. This is because each k-mer is more unique in a local rather than a whole genome assembly. In generating the whole genome assembly, the assembly software (in our case Velvet) aims to identify the region of the genome to which the k-mer corresponds. If this is not possible to determine, contig extension is terminated. In our approach, read-pair information is used to help reduce the number of positions to which a read can be aligned; the search space is therefore reduced and a previously repeated k-mer may become unique in the context of a local assembly. Our results therefore suggest that running IMAGE after each assembly of Illumina data will result in substantial improvements.

In conclusion, we developed and implemented a computational tool that greatly improves draft genome assemblies by utilising high depth of coverage data from second generation sequencing technologies. Our motivation for developing IMAGE was to lower the cost of finishing. Traditional finishing procedures address sequence gaps by designing oligonucleotide primers positioned near contig ends and resequencing selected clones, or obtaining PCR products and sequencing those. In either case, the cost is high compared with random sequencing (by any method). In contrast, IMAGE is fully automated, aligning and inserting the new Illumina contig into the initial assembly, and can be run numerous times to close large gaps. The approach has improved initial assemblies without any manual intervention. It therefore demonstrates the utility of identifying reads for localised reassembly as a cost-effective component of any genome sequencing project.

## Materials and methods

### Algorithm overview

IMAGE is based on two main stages: aligning sequence reads against an initial assembly to identify those that can be used for gap-spanning; and local assembly of the selected subset of reads and updating of the initial assembly by inserting newly assembled contigs to walk into gaps. The two stages can be run repeatedly, producing an improved assembly at each stage for use in the next iteration. Our approach takes advantage of the fact that sequence data from the Illumina GA platform are produced as paired reads from either ends of the same DNA fragment. Each read therefore has a mate-pair and the distance between the two reads can be predicted based on the fragment size in the sequencing library [15]. If a read is aligned uniquely to a contig end but its mate is not aligned anywhere else in the reference, it is likely that the mate resides within the gap where no sequence information yet exists. These reads are pooled and used for contig extension or gap closure as follows (see also Figure 1).

### Alignment and partitioning of Illumina reads

First, contigs and scaffolding information are established in the assembly, which is usually provided by most currently available assemblers or can be obtained by using genome sequences of closely related species as a guide [11]. Illumina sequence reads are then unambiguously mapped onto the assembly using SSAHA2 [16] using parameters suggested in its accompanying manual. SSAHA2 was chosen here as it allows gapped and partial alignment of short reads, but any alignment tool that outputs SAM format [17] can be used interchangeably.

Reads that align at contig ends are partitioned into sets according to which gaps they can span into. If only a single read from a given pair is mapped on these regions, then the mate of this read will be included in the set to which the single read belongs.

#### Local assembly of reads in gapped regions

In our implementation, Velvet (v0.53) [2] was used to assemble sets of Illumina PE reads aligned adjacent to each of the gaps to be spanned. Where scaffolding information is available, reads that are believed to be in the same gap are assembled together. Optimization of Velvet (-exp_cov and -cov_cutoff) was reached by manually inspecting the first few assembled contigs.

#### Iterative extension and merging of contigs

The new contigs are aligned against the reference contig using SSAHA2. In most cases the newly assembled contigs overlap the reference contigs. Depending on the length of the gaps, the contigs in the draft assembly can be extended by the template fragment length of the Illumina reads, or merged if the newly assembled contig aligns against both contigs and supposedly covers the gap. The pipeline can then be run iteratively with the newly inserted contigs as the new 'reference'. Hence, gaps that are longer than the fragment length of Illumina reads can be shortened and closed in subsequent iterations. In some cases, where there are discrepancies between the Illumina contigs and the original assembly, they can be manually inspected quickly using the assembly viewer Gap4 [18].

IMAGE is written in Perl with each stage independent of the other. Hence, different aligners or assemblers can readily replace the default. Because local assemblies are carried out as opposed to a *de novo *one, IMAGE was run successfully in machines with only 6 GB of RAM. IMAGE is available to download at [19].

### Sequence and assembly used in this study

For simulation we used the finished genome sequence of *Salmonella enterica *serovar Paratyphi [GenBank:FM200053]. Three assemblies were produced from the genome sequence of approximately 4.6 Mb. Each assembly contains 30 kb contigs separated by gaps of fixed size (1, 2 or 10 kb). To simulate an ideal Illumina run, 76 nucleotide paired sequences with fragment sizes of 300 bp were derived from the genome sequence with a coverage of 25-fold.

In addition, seven draft genome assemblies were improved with at least two lanes of Illumina reads per assembly using IMAGE. The statistics of these draft assemblies are shown in Table 2. Unless specified in more detail below, the 454 read only assemblies were generated using Newbler, and capillary read assemblies with Arachne [13].

The original draft assembly of the tapeworm *E. multilocularis *was determined by whole-genome shotgun capillary and 454 sequencing. The reads were assembled using Arachne into 2,037 contigs, comprising 106 Mb. From the assembly, a depth of coverage of 7× was calculated. Approximately 120× coverage of Illumina PE 76 bp reads for the assembly were used in IMAGE to improve the assembly. To assess the accuracy of closed gaps, the improved assembly of *E. multilocularis *was then loaded into Gap4 [18]. Here the built in primer selection program OSP [20] was used to design oligonucleotide primers approximately 100 bp away from the 100 contig ends of randomly chosen gaps that were closed previously using our approach. The contig ends were sequenced to span towards the gap and manually aligned into the assembly to assess the accuracy of sequences in the closed gaps.

In the second case study, the genome sequence of the rodent malaria parasite *P. berghei *was determined using various technologies: a separate assembly of two lanes of Illumina 76 bp read pair libraries (mean insert size 130) using Velvet, two 454 (3 kb and 20 kb insert size) runs using Newbler, including approximately 280,000 capillary reads, and finally an assembly of just the capillary reads using Phusion [14]. The contigs were merged and oriented using ABACAS [11] guided by the genome sequence of its closest relative, *P. chabaudi*, rather than doing further hybrid assemblies. The final draft assembly of the core region of the genome consists of 156 gaps within 14 supercontigs. Synteny is not conserved between species in subtelomeric regions; subtelomeres were therefore excluded.

In the final case study, Velvet was used to assemble approximately 274× depth of genome coverage Illumina PE 54 bp reads sequenced from a novel strain of *Salmonella enterica*. The best N50 value was achieved with a k-mer size of 41 bp and -exp_cov parameter set at 70 (Table 2). The 96 supercontigs were then ordered with ABACAS against a reference sequence (*S. typhimurium*; available at [21]) for scaffolding information. All but three supercontigs could be mapped. The final assembly comprises 233 contigs and 233 gaps. As the assembly was generated using k-mer size of 41, we first used the same k-mer parameter to run 5 iterations of IMAGE. Another 5 iterations of IMAGE were run using a k-mer size of 31 before a final set of 3 iterations were run with a k-mer size of 21.

## Abbreviations

bp: base pairs; IMAGE: Iteratively Mapping and Assembly for Gap Elimination; PE: paired end.

## Authors' contributions

IJT, TDO and MB conceived the project and wrote the manuscript. The sequencing project was directed by MB. Assemblies and the IMAGE pipeline were produced by IJT. The data analysis was performed by IJT and TDO. All authors read and approved the final manuscript.

## Supplementary Material

Additional file 1 Comparison of gap closing in the *Echinococcus *assemblies. Click here for file
