# Supplementary material for: Improving draft assemblies by iterative mapping and assembly of short reads to eliminate gaps
Source: Genome Biol. 2010 Apr 13;11(4):R41. doi: 10.1186/gb-2010-11-4-r41 (PMC2884544; doi:10.1186/gb-2010-11-4-r41)
Supplement: Additional file 1 — Comparison of gap closing in the Echinococcus assemblies. [file gb-2010-11-4-r41-S1.doc]

## Supplementary Table 1 - Comparison of gap closing in the *Echinococcus* assemblies

| A) Summary of two *Echinococcus* assemblies | N50 (kb) | Number of contigs | Average  contig length (kb) |
| --- | --- | --- | --- |
| 454/capillary assembly | 108 | 2,310 | 46 |
| Illumina assembly^1^ | 18 | 11,819 | 9 |

^1^ ~120X of Illumina paired end reads was assembled using Abyss and exclude contigs of less than 500bp.

| B) Gaps closed |  |
| --- | --- |
| by *de novo* Illumina assembly | 116 |
| by IMAGE | 895 |
